# A Novel Clinical Score for Differential Diagnosis Between Acute Myocarditis and Acute Coronary Syndrome – The SAlzburg MYocarditis (SAMY) Score

**DOI:** 10.3389/fmed.2022.875682

**Published:** 2022-06-09

**Authors:** Moritz Mirna, Lukas Schmutzler, Albert Topf, Brigitte Sipos, Lukas Hehenwarter, Uta C. Hoppe, Michael Lichtenauer

**Affiliations:** ^1^Division of Cardiology, Department of Internal Medicine II, Paracelsus Medical University, Salzburg, Austria; ^2^Department of Nuclear Medicine and Endocrinology, Paracelsus Medical University, Salzburg, Austria

**Keywords:** cardiology, myocarditis, acute coronary syndrome, clinical score, inflammatory heart disease, score, ACS

## Abstract

**Background:**

Acute myocarditis and acute coronary syndrome (ACS) are important differential diagnoses in patients with new-onset chest pain. To date, no clinical score exists to support the differentiation between these two diseases. The aim of this study was to develop such a score to aid the physician in scenarios where discrimination between myocarditis and ACS appears difficult.

**Materials and Methods:**

Patients with ACS (*n* = 233) and acute myocarditis (*n* = 123) were retrospectively enrolled. Least absolute shrinkage and selection operator (LASSO) regression was conducted to identify parameters associated with the highest or least probability for acute myocarditis. Logistic regression was conducted using the identified parameters and score points for each level of the predictors were calculated. Cutoffs for the prediction of myocarditis were calculated. Validation was conducted in a separate cohort of 90 patients.

**Results:**

A score for prediction of acute myocarditis was calculated using six parameters [age, previous infection, hyperlipidemia, hypertension, C-reactive protein (CRP), and leukocyte count]. Logistic regression analysis showed a significant association between total score points and the presence of myocarditis (*B* = 0.9078, *p* < 0.0001). Cutoff #1 for the prediction of myocarditis was calculated at ≥ 4 (Sens.: 90.3%, Spec.: 93.1%; 46.3% predicted probability for acute myocarditis), cutoff #2 was calculated at ≥ 7 (Sens.: 73.1%, Spec.: > 99.9%; 92.9% pred. prob.). Validation showed good discrimination [area under the curve (AUC) = 0.935] and calibration of the score.

**Conclusion:**

Our clinical score showed good discrimination and calibration for differentiating patients with acute myocarditis and ACS. Thus, it could support the differential diagnosis between these two disease entities and could facilitate clinical decisions in affected patients.

## Introduction

Patients with acute myocarditis and acute coronary syndrome (ACS) often resemble each other in terms of their clinical presentation and diagnostic findings. Because of the possible similarity of both diseases, the European Society of Cardiology (ESC) recommends timely coronary angiography in patients with suspected myocarditis in order to rule out ACS (1, 2). However, a cardiac catheterization laboratory with a physician on-call is, especially in rural areas or developing countries, frequently not available, necessitating inter-hospital transfer accompanied by an emergency care physician (3, 4). Coronary angiography further constitutes an invasive procedure, which is associated with a risk of 1–2% for severe complications, such as allergic reactions to the contrast agent, contrast-induced nephropathy, vascular complications, arrhythmias, peri-procedural myocardial infarction, or cerebrovascular events (5, 6).

Although the risk profiles and comorbidities of patients with acute myocarditis and ACS differ substantially (i.e., age of the patients, presence of classical cardiovascular risk factors, and previous infection), there is, to the best of our knowledge, currently no validated clinical score available to support the differentiation between acute myocarditis and ACS. In compliance with the current recommendations of the ESC, coronary angiography is thus frequently conducted in patients with clinically suspected myocarditis, albeit with a low pretest probability for coronary artery disease. This is especially problematic considering the associated risk of complications, socioeconomic implications, and the significant exposure to radiation during coronary angiography, which is equivalent to 300 conventional chest X-rays (7, 8).

The aim of the current study was to design a score to support the differential diagnosis between myocarditis and ACS, especially in clinical scenarios where differentiation appears difficult, e.g., when patients are neither old nor very young. By strengthening the tentative diagnosis of acute myocarditis, a score could reduce risks and costs associated with potentially avoidable coronary angiography procedures, thus offering benefits in the management of affected patients.

## Materials and Methods

The study was reviewed by the ethical review board of the state of Salzburg, Austria (EK Nr: 1181/2020) prior to enrollment and was conducted in compliance with the Declaration of Helsinki and the principles of Good Clinical Practice. The data supporting the findings of this study are available from the corresponding author upon reasonable request.

### Patients

Eligible patients were identified through a database search on all patients admitted to the University Hospital of Salzburg, Austria, in the time period of 2009–2019. The search was performed for discharge diagnoses, which were classified according to the International Classification of Diseases, Tenth Revision (ICD-10) diagnostic codes (myocarditis: I40.0, I40.1, I40.8, I40.9, and I51.4; ACS: I21.0, I21.1, I21.2, I21.3, I21.4, I21.9, and I24.9). In total, 224 patients with a discharge diagnosis of myocarditis (time period 2009–2019) and 668 patients with a discharge diagnosis of ACS (the year 2019) were identified by database search.

#### Inclusion and Exclusion Criteria

The presence of acute myocarditis or ACS was defined according to the current recommendations of the ESC (1, 9, 10) and was confirmed by revision of all clinical records of identified patients. Patients with myocarditis were included in the subsequent analysis if they fulfilled the criteria for clinically suspected myocarditis by the ESC (1) and had evidence of acute myocarditis on endomyocardial biopsy (EMB) or cardiac magnetic resonance imaging (MRI). Patients with ACS were included if they fulfilled the criteria for ST-segment elevation myocardial infarction (STEMI) or non-ST-segment elevation myocardial infarction (NSTEMI) by the ESC (9, 10). Patients with chronic or recurrent myocarditis, chronic infections (e.g., hepatitis, HIV etc.), or patients admitted for elective procedures were excluded from the study. Thus, 123 patients with acute myocarditis and 233 randomly selected patients with ACS were included in this study.

### Data Acquisition

Demographical data, clinical data, and comorbidities were extracted from the initial hospital record at the presentation. Laboratory data were acquired from the first complete set of laboratory results, i.e., from the emergency department or the intensive care unit.

### Statistical Analyses

Statistical analyses were performed with R [version 4.0.2., R Core Team (11), R Foundation for Statistical Computing, Vienna, Austria^1^ ] using the packages “ggplot2,” “pastecs,” “Hmisc,” “ggm,” “polycor,” “QuantPsyc,” “glmnet,” “ResourceSelection,” and “rms,” as well as SPSS (Version 23.0, IBM, Armonk, NY, United States). Skew and kurtosis of continuous data were assessed visually, and data distribution was assessed by performing a Shapiro–Wilk test. Data were depicted as median ± interquartile range (IQR) and compared using a Mann-Whitney U test since most were not normally distributed. Categorical data were analyzed using Fisher’s exact test.

The cohort was split for the calculation of the score: three-fourths of the data (*n* = 266) were used for score calculation and one-fourth (*n* = 90) was used for validation. The least absolute shrinkage and selection operator (LASSO) regression analysis was conducted to identify parameters associated with the highest or least probability of myocarditis. Logistic regression for the presence of myocarditis was conducted using the identified predictors, Akaike information criterion (AIC) and *R*^2^ were calculated. Average leverage and standardized residuals were calculated to detect relevant outliers, absence of multicollinearity was checked by calculating variance inflation factors (see Table 3). Then, a locally estimated scatterplot smoothing (LOESS) function was performed to identify cut points for numeric variables. Logistic regression analysis was conducted for the newly generated ordinal variables. The total score was calculated for each patient, and binomial logistic regression for the prediction of myocarditis using total score values was performed in the three-fourths cohort. Receiver operating characteristic (ROC) curve and area under the curve (AUC) measurements for the prediction of myocarditis were performed, and cutoff points were defined in the three-fourths of cohort. Cutoff #1 was calculated by means of the Youden index (12), and cutoff #2 was chosen to depict maximum specificity for myocarditis. Validation was performed in the one-fourth validation cohort. A *p*-value of < 0.05 was considered statistically significant.

## Results

### Baseline Characteristics and Comorbidities

A total of 356 patients were enrolled in this study. Of these, 34.6% (*n* = 123) were in the myocarditis subgroup and 65.4% (*n* = 233) in the ACS subgroup. Patients with myocarditis were significantly younger than patients with ACS {median 34 years [interquartile range (IQR) 24 − 44] vs. median 62 years (IQR 55 − 74), *p* < 0.0001}. In both subgroups, the majority of patients was male (myocarditis: 80.5% vs. ACS: 70.8%, *p* = 0.056). Among patients with ACS, 131 patients (56.2%) had STEMI and 102 patients had (43.8%) NSTEMI.

While an infection within the last 4 weeks was significantly more prevalent in patients with myocarditis (66.1 vs. 8.6%, *p* < 0.0001), classical cardiovascular risk factors, such as diabetes mellitus, hyperlipidemia, obesity [body mass index (BMI) > 30 kg/m^2^], hypertension, and history of smoking occurred more frequently in the ACS subgroup. While coronary artery disease, cerebral artery disease, and peripheral artery disease were more prevalent in patients with ACS, there were no statistically significant differences in the frequencies of active malignancies, autoimmune disorders, or anti-inflammatory/immunosuppressive treatments between both groups (see Table 1).

**TABLE 1:** Baseline characteristics of both groups.

	Myocarditis (*n* = 123)		ACS (*n* = 233)		
*Baseline characteristics*	Median	IQR	Median	IQR	*P-value*
Age (years)	34	24–44	62	55–74	< 0.0001

	**%**	**n**	**%**	**n**	***P-value***

Male sex	80.5	99	70.8	165	0.056
Infection within 4 weeks	66.1	80	8.6	20	< 0.0001
Diabetes mellitus	0.8	1	25.2	58	< 0.0001
Hyperlipidemia	16.4	20	73.9	170	< 0.0001
Obesity (BMI > 30 kg/m^2^)	13.8	17	29.1	67	0.002
Arterial hypertension	15.6	19	70.9	163	< 0.0001
History of smoking	33.6	41	48.3	111	0.009
Coronary artery disease	1.6	2	15.7	36	< 0.0001
Cerebral artery disease	0.8	1	14.8	34	< 0.0001
Peripheral artery disease	0	0	6.5	15	0.004
Active malignancy	1.6	2	4.7	10	0.222
Autoimmune disorders	6.5	8	4.7	10	0.616
Immunosuppression	4.1	5	1.9	4	0.298
Corticosteroids	3.9	3	2.6	5	0.690
NSAID	0	0	1.0	2	0.601

*ACS, acute coronary syndrome; IQR, interquartile range; BMI, body mass index; NSAID, non-steroidal anti-inflammatory drugs.*

### Diagnostic Procedures

During hospital stay, coronary angiography, and percutaneous coronary intervention (PCI) were conducted significantly more often in patients with ACS [coronary angiography: 93.6% (*n* = 218) vs. 46.3% (*n* = 57), *p* < 0.0001; PCI: 85.4% (*n* = 187) vs. 0% (*n* = 0), *p* < 0.0001]. In contrast, EMB and MRI were performed more frequently in patients with myocarditis [EMB: 9.9% (*n* = 12) vs. 0% (*n* = 0), *p* < 0.0001; MRI: 98.4% (*n* = 121) vs. 1.7% (*n* = 4), *p* < 0.0001].

### Laboratory Findings

Serum concentrations of C-reactive protein (CRP) were significantly higher in patients with myocarditis [median 40.0 mg/l (IQR 6.0–84.0) vs. median 3.0 mg/l (IQR 1.0–10.0), *p* < 0.0001], whereas peripheral leukocyte counts were higher in patients presented with ACS [11.03 G/l (IQR 8.98–14.00) vs. median 8.50 G/l (IQR 6.76–11.92)]. Furthermore, serum levels of serum creatinine, lactate dehydrogenase (LDH), prothrombin time, and thrombocyte count were significantly higher in patients of the ACS group (see Table 2). Of note, there were no significant differences in the concentrations of high sensitivity troponin (hsTnT), creatinine kinase, or pro-brain natriuretic peptide (pBNP) between patients of both investigated groups.

**TABLE 2:** Laboratory findings in both groups.

	Myocarditis (*n* = 123)		ACS (*n* = 233)		
*Laboratory findings*	Median	IQR	Median	IQR	*P-value*
Creatinine (μmol/l)	79.6	70.7–88.4	88.4	75.2–104.3	0.005
C-reactive protein (CRP) (mg/l)	40.0	6.0–84.0	3.0	1.0–10.0	< 0.0001
Bilirubin (μmol/l)	10.3	6.8–13.7	8.6	5.1–12.0	0.009
Alanine aminotransferase (ALT) (IU/l)	29.0	19.0–42.0	34.5	20.6–55.0	0.169
Aspartate transaminase (AST) (IU/l)	53.0	31.0–76.5	55.0	29.0–107.5	0.284
Lactate dehydrogenase (IU/l)	219	173–271	274	213–414	< 0.0001
Creatinine kinase (CK) (IU/l)	267	132–494	238	120–528	0.898
High sensitivity troponin (hsTnT) (ng/l)	335	50–720	150	52–599	0.326
Pro brain natriuretic peptide (pBNP) (pmol/l)	46.5	21.3–101.5	68.1	13.1–210.7	0.460
Prothrombin time (%)	99	91–104	102	81–115	0.116
Hemoglobin (mmol/l)	9.1	8.5–9.7	8.9	8.3–9.7	0.171
Leukocyte count (G/l)	8.50	6.76–11.92	11.03	8.98–14.00	< 0.0001
Thrombocyte count (G/l)	216	180–258	240	209–274	< 0.0001
Interleukin 6 (pg/ml)	42.80	12.00–69.60	47.75	13.65–61.90	0.945
Procalcitonin (μg/l)	0.20	0.10–0.28	0.10	0.10–0.15	0.170

*ACS, acute coronary syndrome; IQR, interquartile range.*

**TABLE 3:** Logistic regression analysis for the presence of myocarditis using the 6 predictors identified by LASSO regression.

	AIC = 46.11	B	SE	VIF	*P-value*
*Constant*		*1.2132*	*0.0689*		< *0.0001*
Age		–0.0098	0.0012	1.7146	< 0.0001
Infection within 4 weeks		0.2450	0.0458	1.5807	< 0.0001
Hyperlipidemia		–0.2278	0.0391	1.3468	< 0.0001
Hypertension		–0.1387	0.0414	1.5372	< 0.0001
CRP		0.0094	0.0035	1.4152	0.010
Leukocyte count		–0.0213	0.0036	1.0348	< 0.0001

*R^2^ = 0.71 (Hosmer–Lemeshow), 0.15 (Cox-Snell), 0.73 (Nagelkerke). AIC, Akaike information criterion, B, regression coefficient, SE, standard error, VIF, variance inflation factor, BMI, body mass index, CRP, C-reactive protein.*

### Score Calculation

Of a total of 26 parameters, 14 with significant differences between both subgroups were included in the following analysis (age, previous infection, diabetes mellitus, hyperlipidemia, obesity, hypertension, history of smoking, coronary artery disease, peripheral artery disease, cerebral artery disease, serum creatinine, CRP, leukocyte count, and thrombocyte count). LASSO regression analysis identified six of these at λ_1*SE*_ as associated with the highest or least probability of myocarditis (age, previous infection, hyperlipidemia, hypertension, CRP, and leukocyte count; see Supplementary Figure 1). Logistic regression using the six identified predictors is depicted in Table 3.

**FIGURE 1:**
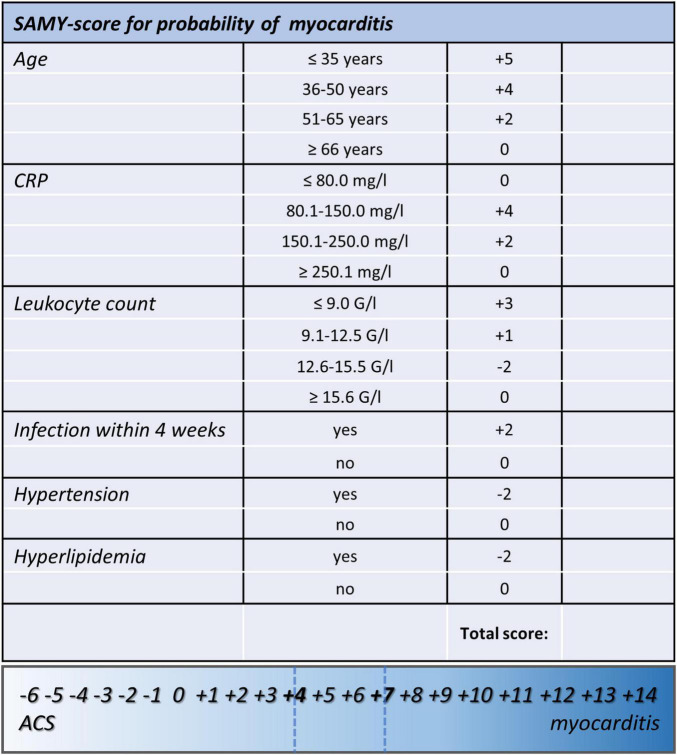
Score sheet of the proposed clinical score for the prediction of myocarditis that includes the two calculated cutoffs (#1: ≥ 4, Sens.: 90.3%, Spec.: 93.1%, PPV: 87.5%, NPV 94.7%; 46.3% predicted probability for myocarditis; #2: ≥ 7, Sens.: 73.1%, Spec.: > 99.9%, PPV: > 99.9%, NPV 87.4%; 92.9% predicted probability for myocarditis). SAMY, SAlzburg MYocarditis score; ACS, acute coronary syndrome; BMI, body mass index; CRP, C-reactive protein.

**FIGURE 2:**
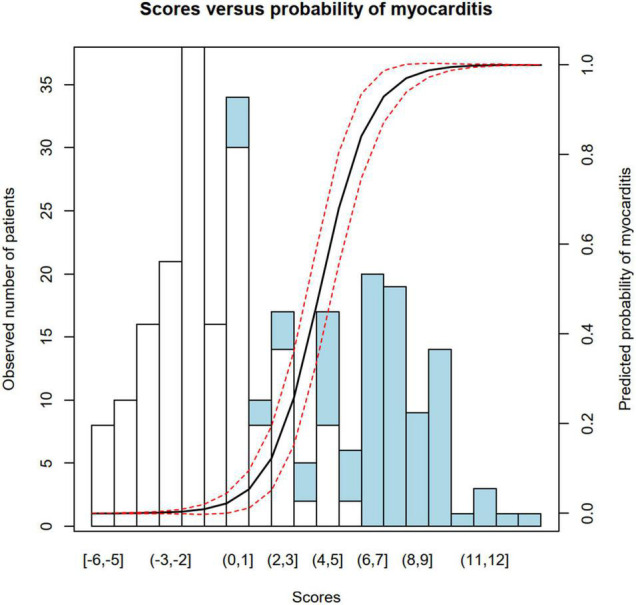
Plot of predicted probability vs. observed values in the three-fourths cohort (*n* = 266).

Locally estimated scatterplot smoothing function was performed to identify cut points for numeric data (age, leukocyte count, and CRP) in order to generate ordinal variables (see Supplementary Figure 2). Then, logistic regression analysis was conducted to identify regression coefficients for dichotomous and ordinal variables, and coefficients were rounded to the nearest integer to create score points for each level of parameters (see Figure 1). The total score was calculated for each patient. Patients in the myocarditis subgroup had significantly higher score values than patients with ACS [median 8 (IQR 6–9) vs. median − 1 (IQR − 2 to 1), *p* < 0.0001]. Predicted probabilities for myocarditis for each sum of score points are depicted in Table 4, a plot of predicted probabilities vs. observed values in the three-fourths cohort is depicted in Figure 2.

**TABLE 4:** Predicted probabilities for myocarditis for each sum of score points.

Score value (points)	Predicted prob. for myocarditis (%)
–6 to –3	< 0.1
–2	0.4
–1	0.9
0	2.2
+1	5.3
+2	12.3
+3	25.8
+4	46.3
+5	68.1
+6	85.0
+7	92.9
+8	97.0
+9	98.8
+10	99.5
+11	99.8
+12 to +14	> 99.9

**FIGURE 3:**
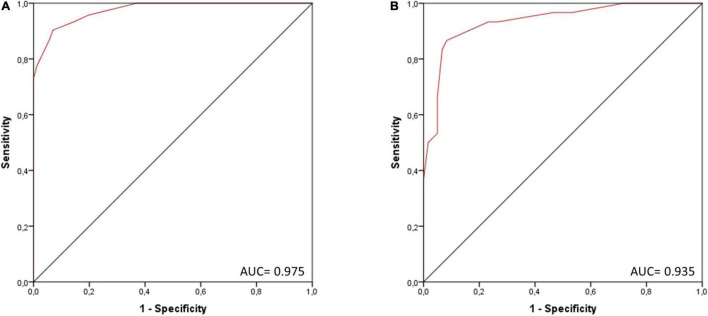
ROC curve of the score for the presence of myocarditis in **(A)** the three-fourths cohort (*n* = 266) and **(B)** the validation cohort (one-fourth of the total cohort, *n* = 90). AUC, area under the curve.

Univariate logistic regression analysis in the three-fourths cohort showed a significant association with the presence of myocarditis [*B* = 0.9078 (SE = 0.1159), *p* < 0.0001; *R*^2^ = 0.71 (Hosmer–Lemeshow), 0.60 (Cox-Snell), 0.83 (Nagelkerke); AIC = 104.83] and adequate goodness-of-fit (*X*^2^ = 5.20, *p* = 0.736). AUC of the score was calculated at 0.975, ROC curve is depicted in Figure 3A. Cutoff #1 was calculated by means of the Youden index (12) (#1: ≥ 4, Sens.: 90.3%, Spec.: 93.1%, positive predictive value (PPV): 87.5%, negative predictive value (NPV) 94.7%; 46.3% predicted probability for myocarditis) and cutoff #2 was calculated to depict optimal specificity for myocarditis (#2: ≥ 7, Sens.: 73.1%, Spec.: > 99.9%, PPV: > 99.9%, NPV 87.4%; 92.9% predicted probability for myocarditis).

Validation of the score in one-fourth cohort (*n* = 90) showed good discrimination (AUC = 0.935; see Figure 3B) and calibration (see Supplementary Figure 3).

## Discussion

New-onset chest pain constitutes a challenging symptom, which requires timely and targeted diagnostic workup (13, 14). In the past, several scoring systems have been implemented in clinical routine to support diagnostic and therapeutic decisions in patients presenting with chest pain. For example, the Wells score and the Geneva score have been developed to facilitate the diagnosis of suspected pulmonary embolism (15, 16), whereas the Aortic Dissection Detection Risk Score (ADD-RS) has been validated to estimate the pretest probability of aortic dissection (17). Similarly, InterTAK Diagnostic Score was developed to support the differentiation between ACS and Takotsubo syndrome (TTS), which frequently resemble each other in terms of clinical presentation, laboratory findings, and abnormalities on electrocardiogram (ECG) and transthoracic echocardiography (TTE) (18).

Similar to TTS, acute myocarditis can mimic ACS. Therefore, current guidelines of the ESC advocate coronary angiography with a class IC recommendation in patients with suspected myocarditis in order to rule out ACS (1, 2). However, in highlight of the risk of complications, the associated exposure to radiation, as well as socioeconomic implications, coronary angiography should only be conducted after strict evaluation of its indication and all risks and benefits for the patient. In addition, patients are often admitted to an intensive care unit for heart rhythm monitoring until ACS is securely ruled out, which is problematic especially in highlight of limited resources during the COVID-19 pandemic (19). In this regard, the absence of a clinical scoring system to support the differential diagnosis between ACS and acute myocarditis becomes apparent, which is why we aimed to develop such a score in this study.

The novel SAlzburg MYocarditis (SAMY) score comprises six clinical parameters, which were selected by LASSO regression due to their high or low probability of myocarditis from a total of 26 possible variables. Interestingly, all of the selected parameters have been associated with the presence of acute myocarditis or ACS in previous studies. As such, acute myocarditis was identified to predominately affect patients of young age and to be associated with a preceding viral infection (1, 20, 21). In contrast, classical cardiovascular risk factors, such as hyperlipidemia or arterial hypertension, are commonly found in patients with ACS (9, 22, 23).

All of the six included parameters can easily be obtained in the emergency department by assessing the patient’s medical history and performing a standard laboratory analysis. Together, they provide a novel clinical score, which estimates the probability of acute myocarditis and shows good discrimination and calibration in our study. Interestingly, of all included predictors, young age was associated with the highest regression coefficients for acute myocarditis, which is depicted by the number of score points per level and is a direct result of the diverse distribution of age between the two investigated groups. Furthermore, patients in both investigated groups showed an increment of CRP and leukocyte count, however, leukocytosis was more pronounced in patients with ACS. Consequently, attribution of score points for CRP and leukocyte count does not follow a linear relationship, but is different for each level of the predictor variable depending on its individual probability for myocarditis (also see Supplementary Figure 2), in order to confer with the well-known and often transient increase of inflammatory biomarkers in patients presented with ACS (24–26).

The novel SAMY score can easily be calculated in the emergency department by using six clinical variables, hereby supporting the differential diagnosis between ACS and acute myocarditis in clinical scenarios where the differentiation between both disease entities appears difficult for the attending physician, e.g., when patients with chest pain are neither old nor very young. However, our score only provides a probability estimate for myocarditis and is not diagnostic *per se*. As such, a low score does not exclude acute myocarditis and a high score is not definitely diagnostic for myocarditis. Nevertheless, it could support the attending physicians in their clinical decisions that may help to allocate resources correctly and to weigh the risks and benefits of coronary angiography for the individual patient.

## Conclusion

Using six clinical parameters, the novel SAMY score provides an estimate of the probability of acute myocarditis in order to support the differentiation between myocarditis and ACS in the acute setting. Hereby, it could help to avoid unnecessary coronary angiography procedures and could support the clinical decisions of the attending physician, especially in clinical scenarios where resources are limited or coronary angiography is not rapidly available.

## Limitations

Because of the low prevalence of myocarditis, we chose a retrospective study design for this study. However, a retrospective design is inferior to a prospective design in terms of the achieved level of evidence. Furthermore, the exclusion of patients without evidence of myocarditis on MRI or EMB greatly reduced the number of enrolled patients, which could have affected statistical analyses, especially regarding the validation of the score in the comparatively small validation cohort (*n* = 90). Therefore, our findings need to be validated in a larger prospective and, preferably multicentric, a study in the future. Moreover, the aim of this study was to create a score to help clinicians differentiate between ACS and acute myocarditis; whether it can be applied to other clinical scenarios or aim in the diagnosis of chronic myocarditis remains to be elucidated in further studies.

## Data Availability Statement

The raw data supporting the conclusions of this article will be made available by the authors, without undue reservation.

## Ethics Statement

The studies involving human participants were reviewed and approved by the Ethical Review Board of the state of Salzburg, Austria (EK Nr: 1181/2020). Written informed consent for participation was not required for this study in accordance with the national legislation and the institutional requirements.

## Author Contributions

MM and ML concepted and designed the study. LS performed the data collection. MM conducted the statistical analyses. MM, AT, BS, and LS wrote the manuscript. UH, LH, and ML reviewed the manuscript and provided substantial improvements prior to submission. All authors read the final version of the manuscript and agreed to its contents.

## Conflict of Interest

The authors declare that the research was conducted in the absence of any commercial or financial relationships that could be construed as a potential conflict of interest.

## Publisher’s Note

All claims expressed in this article are solely those of the authors and do not necessarily represent those of their affiliated organizations, or those of the publisher, the editors and the reviewers. Any product that may be evaluated in this article, or claim that may be made by its manufacturer, is not guaranteed or endorsed by the publisher.

## Footnotes

1
http://www.R-project.org/

## Abbreviations

ACS: acute coronary syndrome
ADD-RS: Aortic Dissection Detection Risk Score
AIC: Akaike information criterion
AUC: area under the curve
BMI: body mass index
CRP: C-reactive protein
ECG: electrocardiogram
EMB: endomyocardial biopsy
ESC: European Society of Cardiology
HIV: human immunodeficiency virus
hsTnT: high sensitivity troponin
IQR: interquartile range
LASSO: least absolute shrinkage and selection operator
LDH: lactate dehydrogenase
LOES: locally estimated scatterplot smoothing
MRI: magnetic resonance imaging
NSAID: non-steroidal anti-inflammatory drug
NSTEMI: non-ST-segment elevation myocardial infarction
pBNP: pro brain natriuretic peptide
PCI: percutaneous coronary intervention
ROC: receiver operating characteristic
SAMY: SAlzburg MYocarditis score
SE: standard error
STEMI: ST-segment elevation myocardial infarction
TTE: transthoracic echocardiography
TTS: Takotsubo syndrome
VIF: variance inflation factor.
