# Supplementary material for: A Novel Clinical Score for Differential Diagnosis Between Acute Myocarditis and Acute Coronary Syndrome – The SAlzburg MYocarditis (SAMY) Score
Source: Front Med (Lausanne). 2022 Jun 9;9:875682. doi: 10.3389/fmed.2022.875682 (PMC9218572; doi:10.3389/fmed.2022.875682)
Supplement: Supplementary file 1 [file Data_Sheet_1.pdf]

A novel clinical score for differential diagnosis between acute myocarditis and acute coronary syndrome – the SAlzburg MYocarditis (SAMY) score

-Supplemental materials-

Moritz MIRNA, MD, PhD[1], Lukas SCHMUTZLER[1], Albert TOPF, MD[1], Brigitte SIPOS, MD[1], Lukas HEHENWARTER, MSc[2], Prof. Uta C. HOPPE, MD[1], Assoc.Prof. Michael LICHTENAUER, MD, PhD[1]

[1] Department of Internal Medicine II, Division of Cardiology, Paracelsus Medical University of Salzburg, Austria

[2] Department of Nuclear Medicine and Endocrinology, Paracelsus Medical University of Salzburg, Austria

Running Head: Clinical score for myocarditis.

Correspondence:

Moritz MIRNA, MD, PhD

Department of Internal Medicine II
Division of Cardiology
Universitätsklinikum der Paracelsus Medizinischen Universität
Müllner Hauptstraße 48
5020 Salzburg
Austria

e-Mail: m.mirna@salk.at

Telephone: +43 (0) 57255 - 58340

ORCID iD: https://orcid.org/0000-0001-5679-4872

Supplemental Figure I:

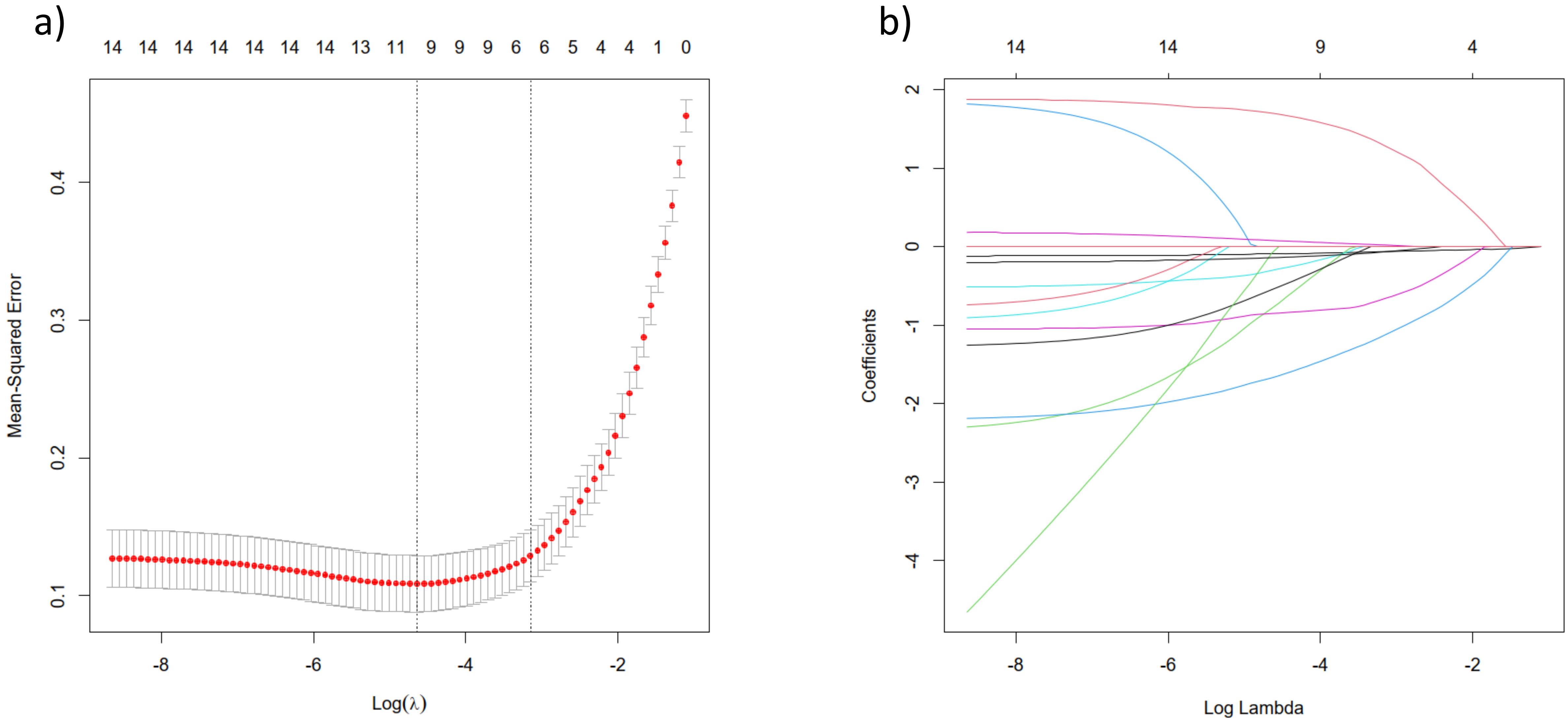

Suppl. Figure I: a) Cross-validation plot of LASSO regression. Depicted are Mean-Squared Error (MSE), log(λ) and the number of non-zero regression coefficients in the model. Vertical lines depict the log(λ) of λ_{min} and λ_{1SE}. b) variable coefficients plotted against log(λ) and the number of non-zero regression coefficients in the model.

Supplemental Figure II:

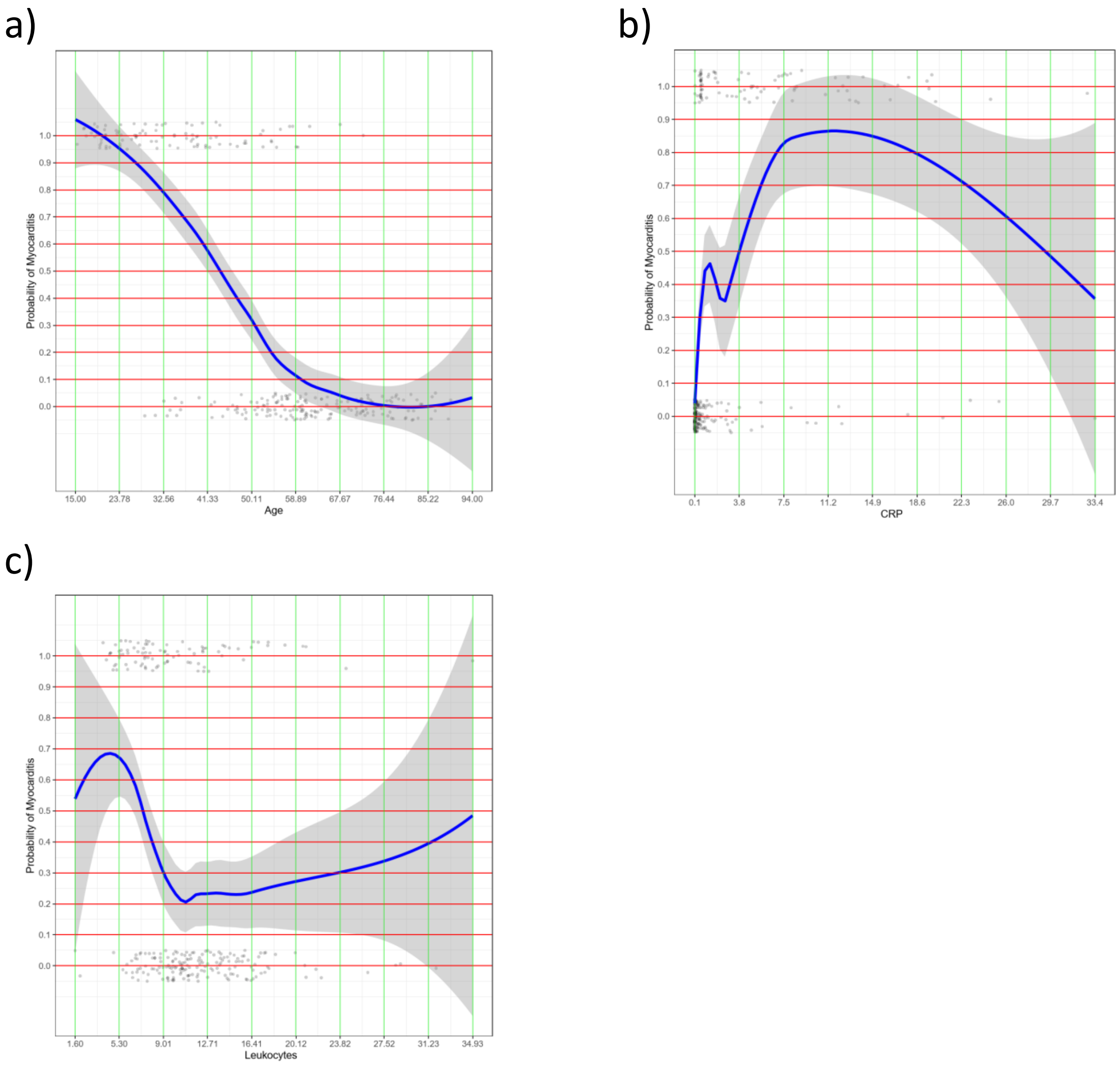

Suppl. Figure II: Scatterplots of continuous data against probability of myocarditis using locally estimated scatterplot smoothing (LOESS) function: a) Age (years), b) C-reactive protein (CRP; mg/l), c) Leukocyte count (G/l)

Supplemental Figure III:

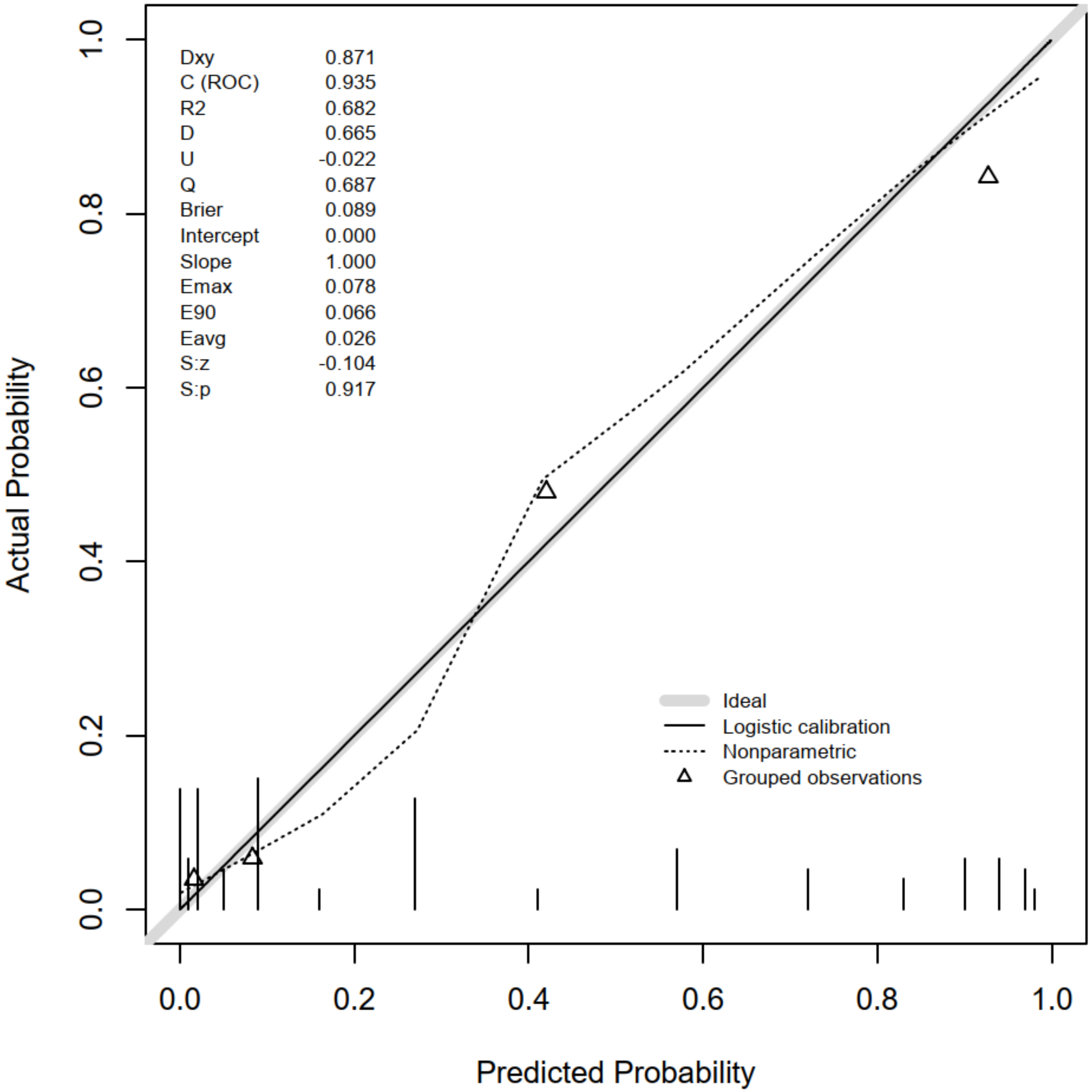

Suppl. Figure III: Validation plot in the validation cohort (¼ of total cohort, n= 90). Depicted are actual and predicted probability and measures of calibration (D_{xy}= Somers' D_{xy} rank correlation, C(ROC)= receiver operating characteristic, R2= Nagelkerke-Cox-Snell-Maddala-Magee R-squared index, D= Discrimination index, U= Unreliability index, Q= Quality index, Brier score, s:z= Spiegelhalter z-test for calibration accuracy and its two-tailed p-value).
